# Trends for Neonatal Deaths in Nepal (2001–2016) to Project Progress Towards the SDG Target in 2030, and Risk Factor Analyses to Focus Action

**DOI:** 10.1007/s10995-019-02826-0

**Published:** 2019-11-26

**Authors:** Ashish Kc, Anjani Kumar Jha, Mahendra Prasad Shrestha, Hong Zhou, Abhishek Gurung, Jeevan Thapa, Shyam Sundar Budhathoki

**Affiliations:** 1 Department of Women’s and Children’s Health, International Maternal and Child Health, University Hospital, Uppsala University, 751 85 Uppsala, Sweden; 2 Nepal Health Research Council, Kathmandu, Nepal; 3 Ministry of Health and Population, Government of Nepal, Kathmandu, Nepal; 4 Department of Maternal and Child Health, School of Public Health, Peking University, Beijing, China; 5 Golden Community, Lalitpur, Nepal; 6 School of Public Health and Community Medicine, B.P. Koirala Institute of Health Sciences, Dharan, Nepal

**Keywords:** Neonatal health, Equity, Multi-sectoral interventions, Every Newborn Action Plan, Sustainable development goals

## Abstract

**Introduction:**

Nepal has made considerable progress on improving child survival during the Millennium Development Goal period, however, further progress will require accelerated reduction in neonatal mortality. Neonatal survival is one of the priorities for Sustainable Development Goals 2030. This paper examines the trends, equity gaps and factors associated with neonatal mortality between 2001 and 2016 to assess the likelihood of Every Newborn Action Plan (ENAP) target being reached in Nepal by 2030.

**Methods:**

This study used data from the 2001, 2006, 2011 and 2016 Nepal Demographic and Health Surveys. We examined neonatal mortality rate (NMR) across the socioeconomic strata and the annual rate of reduction (ARR) between 2001 and 2016. We assessed association of socio-demographic, maternal, obstetric and neonatal factors associated with neonatal mortality. Based on the ARR among the wealth quintile between 2001 and 2016, we made projection of NMR to achieve the ENAP target. Using the Lorenz curve, we calculated the inequity distribution among the wealth quintiles between 2001 and 2016.

**Results:**

In NDHS of 2001, 2006, 2011 and 2016, a total of 8400, 8600, 13,485 and 13,089 women were interviewed respectively. There were significant disparities between wealth quintiles that widened over the 15 years. The ARR for NMR declined with an average of 4.0% between 2001 and 2016. Multivariate analysis of the 2016 data showed that women who had not been vaccinated against tetanus had the highest risk of neonatal mortality (adjusted odds ratio [AOR] 3.38; 95% confidence interval [CI] 1.20–9.55), followed by women who had no education (AOR 1.87; 95% CI 1.62–2.16). Further factors significantly associated with neonatal mortality were the mother giving birth before the age of 20 (AOR 1.76; CI 95% 1.17–2.59), household air pollution (AOR 1.37; CI 95% 1.59–1.62), belonging to a poorest quintile (AOR 1.37; CI 95% 1.21–1.54), residing in a rural area (AOR 1.28; CI 95% 1.13–1.44), and having no toilet at home (AOR 1.21; CI 95% 1.06–1.40). If the trend of neonatal mortality rate of 2016 continues, it is projected that the poorest family will reach the ENAP target in 2067.

**Conclusions:**

Although neonatal mortality is declining in Nepal, if the current trend continues it will take another 50 years for families in the poorest group to attain the 2030 ENAP target. There are different factors associated with neonatal mortality, reducing the disparities for maternal and neonatal care will reduce mortality among the poorest families.

**Electronic supplementary material:**

The online version of this article (10.1007/s10995-019-02826-0) contains supplementary material, which is available to authorized users.

## Significance

Sustainable development goals (SDG) 3.2 sets the target of reducing the neonatal mortality rate to 12 or less per 1000 live births by 2030. This paper uses the data from four nationally representative surveys to map the trends and annual rate of reduction for neonatal mortality in Nepal. Between 2001 and 2016, the neonatal mortality rate decreased, however, the disparities between the poor and rich have widened. A multivariate analysis was carried out to identify the socio-demographic, maternal, obstetric and neonatal factors associated with neonatal mortality in Nepal. Reducing neonatal mortality rate among the poor families will require a reducing disparity for care.

## Ethics

The NDHS received ethical approval from the Nepal Health Research Council. Verbal consent was taken from interviewees during the DHS interviews. Approval was obtained from MEASURE DHS, ICF Macro to use the NDHS data for the current study.

## Introduction

The Millennium Development Goals (MDGs) placed maternal and child health at front and centre of the global health agenda resulting in unprecedented attention and investment in reproductive, maternal and child health (UNICEF 2019; United Nations 2001) Globally, the under-five mortality rate (MDG 4) reduced by 52% and the maternal mortality ratio (MDG 5) by 30% over the MDG period (1990–2015) (Kassebaum et al. 2016; Wang et al. 2016).

Towards the end of the MDG era in 2014, there was a global call for action for reducing neonatal mortality through the Every Newborn Action Plan (ENAP) (World Health Organisation 2014). One of the 13 SDG targets for health (SDG 3.2) was to reduce the national neonatal mortality rate (NMR) to 12 or less per 1000 live births by 2030 (United Nations 2015).

In 2014 in Nepal, 13,000 neonates died, which was 61% of all under-5 deaths (Lawn et al. 2014; Ministry of Health 2017). To further accelerate reduction in neonatal mortality, investment was pledged by the government and its development partners under Nepal’s Every Newborn Action Plan (NENAP) (Ministry of Health and Population 2016). Nepal has pledged to reduce the 2015 neonatal mortality rate of 23 per 1000 live births, to 12 per 1000 live births by 2030. However, the further reduction of neonatal mortality will require a thorough review of the trends in the mortality rate by socioeconomic strata and the identification of the factors associated with neonatal mortality. In the last 15 years in Nepal, evidence-based advocacy has led to newborn health being a major focus of the MDGs (Pradhan et al. 2012; Khatri et al. 2016). Policies were formulated and adopted to make newborn health a national issue and an integral part of maternal and child health programmes (Kc et al. 2011). The Government of Nepal has promoted newborn health in partnership with developmental partners, academia and professional associations (Ministry of Health and Population, Nepal 2016).

Based on lessons learned from implementing community and health facility-based newborn health programmes, Nepal developed an approach to newborn health care that was in line with the local context. The Government of Nepal has focused on rolling out a training packages for integrated management of neonatal and childhood illness (IMNCI) and Skilled Birth Attendance (Ministry of Health and Population, Nepal 2006, 2007, 2015) along with quality improvement interventions to use in health institutions (Kc et al. 2017). Despite the progress made in maternal and neonatal health, the disparities between the socio-economic group has widened (Målqvist et al. 2017).

This paper examines trends, equity gap and factors associated with neonatal mortality between 2001 and 2016 in Nepal to project the neonatal mortality rate in 2030 and when the Every Newborn Action Plan (ENAP) target will be achieved.

## Methods

This study carried out a secondary analysis of data from the nationally representative cross sectional 2001, 2006, 2011 and 2016 Nepal Demographic and Health Surveys (NDHS) (Ministry of Health, Nepal et al. 2002, 2007, 2012, 2017). These surveys used household, women’s and men’s questionnaires to gather demographic and health data. This study used the data from the women’s questionnaire, which was administered to women age 15–49 years. The questionnaires were based on the standard MEASURE DHS questionnaires. NDHS surveys use national population census data as the basis for their sampling frames. Probability proportionate to size sampling was used to recruit a nationally representative sample of respondents. A response rate of more than 90% was achieved in all four surveys (Web-Appendix).

### Variables

The datasets were extracted in SPSS version 23.0. For this study, we extracted the following variables from the dataset.

Neonatal mortality: Defined probability of dying within the first month of life,

Socio-demographic factors:

- Wealth quintile—the NDHS used wealth quintiles based on the World Bank categories of poorest, poorer, middle, richer and richest quintiles (Filmer and Pritchett 2001; Rutstein and Johnson 2004),
- Place residence—urban or rural,
- Administrative region—seven provinces,
- Ecological region—mountain, hill and tarai,
- Availability of toilet at home—yes or no.

Maternal factors:

- Level of maternal education—uneducated (no formal education) and education (primary education or more),
- Mother’s chewing tobacco—yes or no,
- Smokes cigarette at home—yes or no,
- Maternal age categorized in 5-year interval—15–19 years, 20–24 years, 25–29 years, 30–34 years, 35–39 years, 40–44 years and 45–49 years,
- Interval since previous birth— > 48 months, 36–48 months, 24–36 months, < 24 months.

Obstetric factors:

- Age of first pregnancy categorized into less than 20 years and 20 years and more,
- Duration of pregnancy—less than 9 months and 9 months or more,
- Antenatal care visit—less than 4 visit and 4 visit or more,
- Tetanus vaccination received during pregnancy—yes or no,
- Place of delivery—home or health institution,
- Use of clean delivery kit in home delivery—yes or no.

Neonatal factors:

- Timing of baby put to breast after birth—within 1 h or more than 1 h,
- Birth weight-less than 2500 g and 2500 g or more,
- Sex of the baby at birth—female or male,
- Subjective assessment of the mother about child-size after birth (1 = average; 2 = smaller than average; 3 = larger than average),
- Birth in last 3 years—yes or no,
- Postnatal check up by skilled health worker or non-skilled health worker or traditional worker.

All descriptive and inferential statistics involved weighted analysis. Complex sample analysis was done to test associations considering sample domain, cluster size and sample weight.

Variables with more than two categories were recoded into dichotomous variables for the purpose of bivariate and multivariate analysis.

### Data Analysis

We analysed trend in neonatal mortality by socio-demographic characteristics (wealth quintile and residence), maternal factor (education and age), obstetric factor (interval since previous birth and birth order) and neonatal factor (sex and maternal perception on size at birth).

Based on the neonatal mortality trend, the study generated annual rates of reduction (ARR) in neonatal mortality for the three five-year intervals between the four NDHSs and an overall 15 years’ average. ARR is used for the analysis for monitoring and evaluation of the trend in neonatal mortality, to quantify the rate of change of the prevalence from baseline to the current year. Annual rate of reduction (ARR) based on the following equation:

$$ ARR = - \left( { \left( {\frac{NMRt2}{NMRt1}} \right)^{{\frac{1}{t2 - t1}}} - 1} \right) \times 100 . $$

In the above equation t_2_ is the end year and t_1_ the beginning year. An ARR from 2001–2006 would therefore be calculated as, ((NMR_2006_/NMR_2001_)^(1/5)^) − 1) × 100. We analysed the ARR by socio-demographic factors (wealth quintile and residence), maternal factors (education and age), obstetric factors (interval since previous birth and birth order) and neonatal factor (sex and maternal perception of size of babies).

We assessed the association between the socio-demographic, maternal, obstetric and neonatal factor with neonatal mortality. Tests of association between neonatal mortality and socio-demographic factors were carried out using Pearson’s Chi square test and the student *t* test. The variables with p values < 0.1 were considered for multivariate analysis. The variables were checked for collinearity and the collinear variables were excluded from the multivariate analysis. Collinearity was found between duration of pregnancy and birth weight, this was excluded from the multi-variate analysis. The samples with missing values for the variables considered in the multivariate analysis were excluded.

The Lorenz curve was generated and Gini coefficient calculated to measure the differences in neonatal mortality between the different wealth quintiles. The Gini coefficient ranges between 0 and 1, with 0 denoting perfectly equal distribution and 1 representing perfect inequity. Inequity is represented by the area between the line of perfect distribution and the Lorenz curve. Therefore, the greater the area between the line of perfect distribution and the Lorenz curve, the greater the inequity of distribution.

Based on the ARR for different socioeconomic groups, i.e., wealth quintile for the 15 years between 2001 and 2016, projection on neonatal mortality were calculated up to 2067.

## Results

In 2001, 2006, 2011 and 2016 a total of 8400, 8600, 13,485 and 13,089 women were interviewed respectively (Web-Appendix).

### Trend in Neonatal Mortality

The examination of the neonatal mortality rate across the socioeconomic strata for the 2001–2016 period shows significant disparities between different population groups, and that all these disparities either widened or remained constant over the 15-year period. In 2001, the NMR was one and a half times higher among infants born into the poorest quintile compared to those born into the richest quintile; while in 2016 infants in the poorest quintile were three times more likely to die than infants born into the wealthiest quintile. This reflects the much larger decrease in the NMR among wealthy families. The NMR of families with uneducated or only primary school-educated mothers remained double the NMR of families with educated women in 2001 and 2016, although the NMRs fell for both groups over this period. The NMR was higher in male babies than female babies. The risk of neonatal mortality in babies who were born at below average size was higher at all measurement points than larger babies, with the disparity decreasing a little in 2016. Among healthcare factors, the babies of women who lacked adequate antenatal care and did not deliver at a health institution had a higher neonatal mortality rate (Web-Appendix).

### Trend in ARR of Neonatal Mortality

The ARR for neonatal mortality was 4.0% between 2001 and 2016, there was a slower ARR between 2001 and 2006 (3.0%), with no ARR between 2006 and 2011 and accelerated by 8.6% between 2011 and 2016. Among the different quintiles, the ARR for neonatal mortality was highest among the wealthiest (6.3%) and slowest among the poorest (2.0%) between 2001 and 2016. The ARR was higher in families residing in urban areas (3.7%) than the families residing in rural areas between 2001 and 2016. The ARR for neonatal mortality was highest among the women who delivered at an interpregnancy interval of 24–36 months (8.9%) between 2001 and 2016. The ARR for neonatal mortality was higher among the female babies (5.0%) than the male babies (3.0%) and among babies who had an average or above weight at birth (4.1%) than small or very small babies (3.3%) between 2001 and 2016 (Table 1).

**Table 1:** Trend in annual rate of reduction in neonatal mortality rate by socio-demographic characteristics (per 1000 live births, 2001–2016 NDHSs)

	2001–2006	2006–2011	2011–2016	2001–2016
Annual rate of reduction of NMR	3.3	0.0	8.6	4.0
Wealth quintile				
Poorest	2.6	3.0	0.5	2.0
Second poorest	7.5	− 1.0	3.8	3.5
Middle	0.8	3.7	7.8	4.1
Second richest	8.0	− 3.6	11.6	5.5
Richest	4.1	6.1	8.8	6.3
Place of residence				
Urban	7.5	0.0	3.4	3.7
Rural	4.0	2.1	1.7	2.6
Mother’s level of education				
No education or only primary	3.4	1.5	3.3	2.7
Secondary or higher	3.4	− 4.4	9.3	2.9
Maternal age in completed years				
< 20	5.0	1.5	5.2	3.9
20–29	4.4	0.0	8.1	4.2
30–39	3.5	5.6	− 2.8	2.2
40–49	13.8	5.3	− 12.0	3.0
Interval since previous birth				
> 48 months	12.9	8.3	3.3	8.2
36–48 months	13.2	9.3	− 1.0	7.4
24–36 months	11.5	7.5	7.8	8.9
< 24 months	13.7	0.0	11.4	8.6
Birth order of infant				
1	4.1	0.9	7.8	4.3
2	8.0	− 0.7	8.7	5.4
3	0.0	4.5	1.3	2.0
4	3.8	4.6	− 19.3	− 3.1
Sex of infant				
Male	5.6	1.0	2.3	3.0
Female	3.0	2.3	9.5	5.0
Size of infant at birth				
Average or larger	3.3	− 1.4	10.1	4.1
Small or very small	0.7	1.9	7.3	3.3

### Risk Factor for Neonatal Mortality

A multivariate analysis of all the variables with a bivariate analysis p value of < 0.1 showed that the wealth status of a family, place of residence, province and ecological zone of residence, and ethnicity were associated with neonatal mortality (Table 2). In 2016, women who had not received a tetanus vaccination had the highest risk of neonatal mortality (AOR 3.38; 95% CI 1.20–9.55), followed by women with no education (AOR 1.87; 95% CI 1.62–2.16). Other factors associated with neonatal mortality in 2016 were giving birth before the age of 20 (AOR 1.76; CI 95% 1.17–2.59), household air pollution (AOR 1.37; CI 95% 1.59–1.62), belonging to a poorest quintile (AOR 1.37; CI 95% 1.21–1.54), residing in a rural area (AOR 1.28; CI 95% 1.13–1.44), and having no toilet at home (AOR 1.21; CI 95% 1.06–1.40) (Table 3).

**Table 2:** The association of socio-demographic, maternal, obstetric and neonatal factors with neonatal mortality in 2016 NDHS

	Neonatal mortality		p-value
	No n (%)	Yes n (%)	
Socio-demographic factor			
Wealth quintile			
Poorest	5167 (94.5)	303 (5.5)	< 0.01*
Second poorest	5098 (94.7)	287 (5.3)	
Middle	5262 (95.0)	279 (5.0)	
Second richest	4762 (96.4)	179 (3.6)	
Richest	3970 (97.5)	103 (2.5)	
Total	24,259 (95.5)	1151 (4.5)	
Place of residence			
Urban	13,869 (96.0)	571 (4.0)	< 0.01*
Rural	10,390 (94.7)	580 (5.3)	
Total	24,259 (95.5)	1151 (4.5)	
Region			
Province 1	3764 (96.9)	119 (3.1)	< 0.01*
Province 2	6044 (94.9)	326 (5.1)	
Province 3	4060 (96.5)	149 (3.5)	
Province 4	2109 (96.5)	74 (3.4)	
Province 5	4272 (94.7)	240 (5.3)	
Province 6	1640 (94.5)	96 (5.5)	
Province 7	2370 (94.2)	147 (5.8)	
Total	24,259 (95.5)	1151 (4.5)	
Ecological zone			
Mountain	1645 (94.0)	105 (6.0)	< 0.01*
Hill	9617 (96.1)	391 (3.9)	
Tarai	12,997 (95.2)	655 (4.8)	
Total	24,259 (95.5)	1151 (4.8)	
Toilet facility at home			
No	4678 (94.0)	300 (6.0)	< 0.01*
Yes	18,927 (95.8)	820 (4.2)	
Total	23,605 (95.5)	1120 (4.5)	
Maternal factor			
Level of education of mother			
Uneducated	13,745 (94.3)	838 (5.7)	< 0.01*
Educated	10,514 (97.1)	313 (2.9)	
Total	24,259 (95.5)	1151 (4.5)	
Mother chews tobacco			
No	23,295 (95.6)	1083 (4.4)	< 0.01*
Yes	964 (93.4)	68 (6.6)	
Total	24,259 (95.5)	1151 (4.5)	
Smokes cigarettes at home			
No	21,805 (95.6)	998 (4.4)	< 0.01*
Yes	2454 (94.1)	153 (5.9)	
Total	24,259 (95.5)	1151 (4.5)	
Age in 5-year groups			
15–19	382 (96.5)	14 (3.5)	< 0.01*
20–24	1950 (96.3)	75 (3.7)	
25–29	3865 (97.2)	112 (2.8)	
30–34	4458 (95.9)	193 (4.1)	
35–39	4743 (95.3)	232 (4.7)	
40–44	4625 (94.5)	267 (5.5)	
45–49	24,258 (95.5)	1151 (4.5)	
Total	13,869 (96.0)	571 (4.0)	
Age of first pregnancy in years			
< 20	14,060 (94.9)	751 (5.1)	< 0.01*
≥ 20	10,199 (96.2)	400 (3.8)	
Total	24,259 (95.5)	1151 (4.5)	
Obstetric factors			
Antenatal care visit			
< 4	1196 (97.9)	28 (2.3)	< 0.01*
≥ 4	2753 (99.3)	20 (2.3)	
Total	3949	48	
Tetanus vaccination received during pregnancy			
No	286 (96.7)	10 (3.3)	< 0.01*
Yes	3636 (98.9)	39 (1.1)	
Total	3925 (98.8)	49 (1.2)	
Duration of pregnancy			
< 9 months	318 (86.9)	48 (13.1)	< 0.01*
≥ 9 months	5122 (98.4)	85 (1.6)	
Total	5440 (97.6)	133 (2.4)	
Place of delivery			
Home	2037 (97.2)	59 (2.8)	< 0.01*
Health institution	2686 (98.3)	46 (1.7)	
Total	4723 (97.8)	105 (2.2)	
Use of clean delivery kit in home deliveries			
No	1357 (98.3)	23 (1.7)	0.805
Yes	299 (98.7)	4 (1.3)	
Total	1656 (98.4)	27 (1.3)	
Neonatal factors			
Timing of baby put to breast after birth			
≥ 1 h	2860 (99.4)	16 (0.6)	0.186
< 1 h	1053 (99.1)	10 (0.9)	
Total	3913 (99.1)	26 (0.9)	
Birth weight			
Less than 2500 g	371 (95.5)	12 (4.5)	0.253
≥ 2500 g	4574 (96.9)	104 (3.1)	
Total	4945 (97.7)	116 (2.2)	
Sex of child			
Male	12,385 (94.7)	693 (5.3)	< 0.01*
Female	11,874 (96.3)	458 (3.7)	
Total	24,259 (95.5)	1151 (4.5)	
Size of baby at birth			
Large and above	794 (96.4)	30 (3.6)	< 0.01*
Average	3314 (98.5)	50 (1.5)	
Small	830 (96.4)	31 (3.6)	
Total	4938 (97.8)	111 (2.2)	
Birth in last 3 years			
No birth	18,385 (95.3)	911 (4.7)	< 0.01*
Yes birth	5874 (96.1)	240 (3.9)	
Total	24,259 (95.5)	1151 (4.5)	
Postnatal check up			
By skilled health worker	1293 (99.4)	8 (0.6)	
By non-skilled health worker	156 (99.4)	1 (0.6)	
By traditional/other	24 (100)	0 (0)	
Total	1473 (99.4)	9 (0.6)	

*Denotes statistically significant between the population group

**Table 3:** Associations between socio-demographic, maternal, obstetric and neonatal factors with neonatal mortality in the 2016 NDHS (multi-variate analysis)

Factors	AOR (95% CI)
Age at first pregnancy less than 20 years	1.76 (1.17–2.59)
No tetanus immunization	3.38 (1.20–9.55)
No education	1.87 (1.62–2.16)
No toilet	1.21 (1.06–1.40)
Household air pollution	1.37 (1.59–1.62)
Rural residence	1.28 (1.13–1.44)
Poor family	1.37 (1.21–1.54)

*AOR* adjusted odds ratio, *CI* confidence interval

### Inequity in Neonatal Mortality

**Fig. 1:**
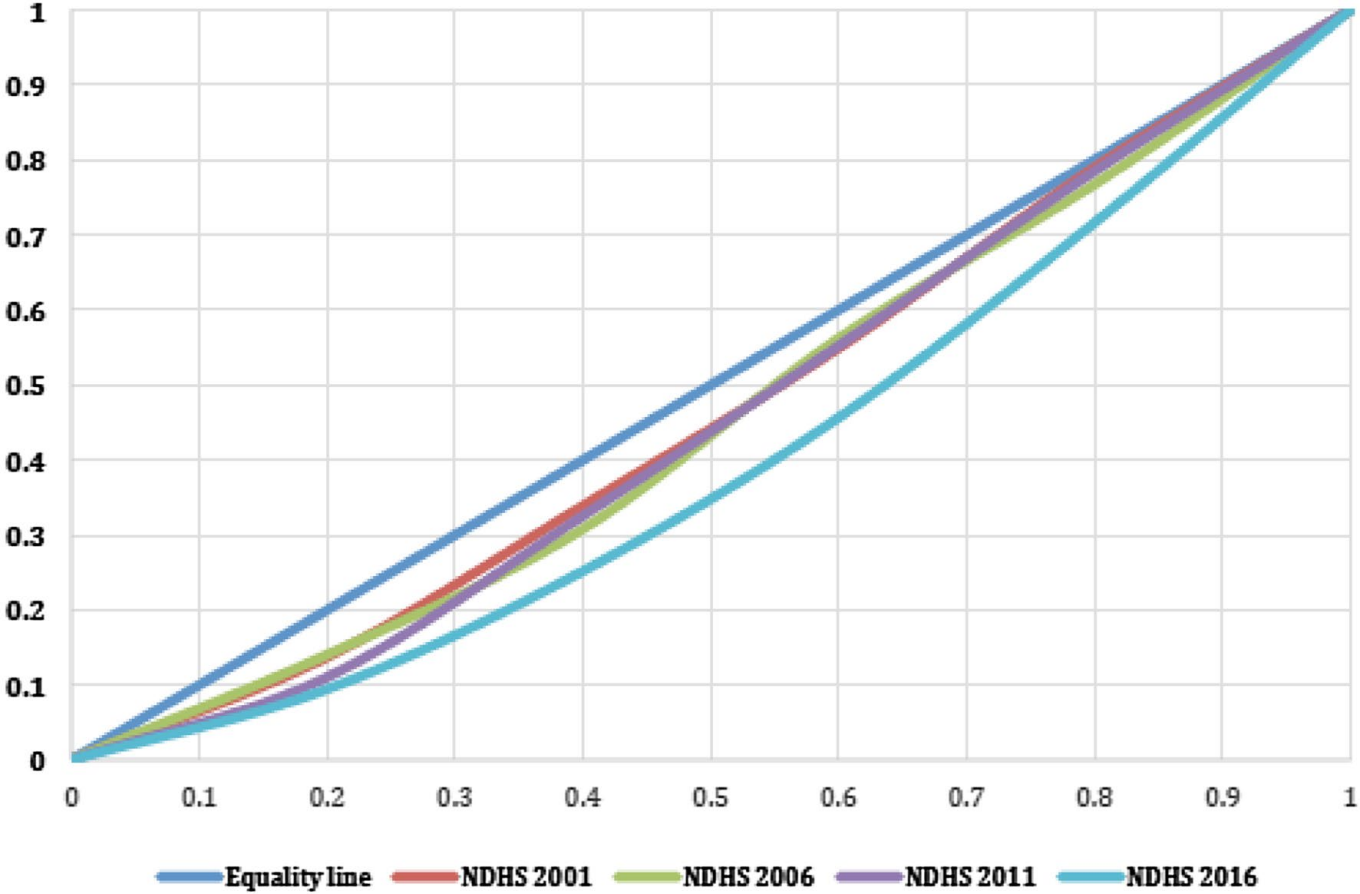
Gini coefficient and inequity gap in neonatal mortality by wealth quintile in the 2001, 2006, 2011 and 2016 NDHSs

From 2001 to 2016, the inequity gradient among the wealth quintiles increased. Using the Lorenz curve and the Gini coefficient, the inequity gap on neonatal mortality between the poorest and wealthiest families was lowest in 2001 and increased over time through 2016 (Fig. 1).

### Projection of Neonatal Mortality Rate

**Fig. 2:**
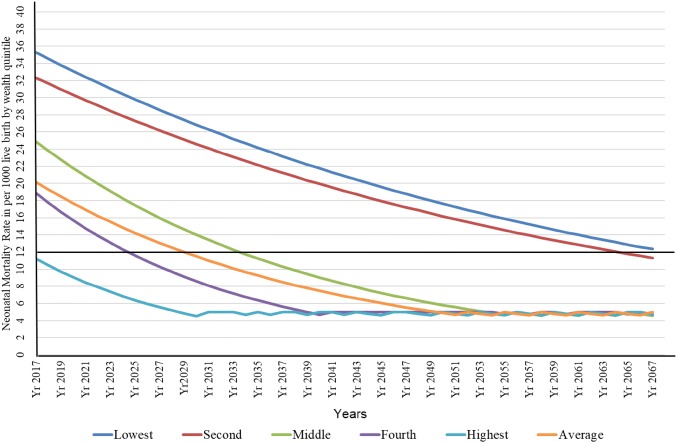
Projection of neonatal mortality by wealth quintile based on the current annual rate of reduction (ARR) (2017 to 2067 AD)

The NMR in the wealthiest quintile achieved the ENAP target of 12 per 1000 live births in 2016. Based on the ARR between 2001 and 2016, the projection for neonatal mortality among the wealthiest quintile group showed the ENAP target will be met at different time. The poorest quintile will reach the ENAP target in 2067, next poorest quintile will reach the ENAP target in 2063 (Fig. 2).

## Discussion

Neonatal mortality in Nepal reduced considerably from 2001 to 2016, with an accelerated rate of reduction between 2010 and 2015. However, there was a greater reduction among the wealthiest families and to babies born by educated women. Inequities have therefore widened. The highest risk of babies dying in their first 28 days was among the poorest families and babies born to uneducated mothers. There was also a greater risk of neonatal death in families that did not have a toilet and for women who smoked or chewed tobacco. Among healthcare factors, the babies of women who lacked adequate antenatal care and did not deliver at a health institution had a higher neonatal mortality rate.

The neonatal projection indicates that, if the current trajectory for neonatal mortality reduction continues, the country will reach the ENAP and SDG target for NMR before 2030. However, this will happen alongside a large disparity between the different socioeconomic groups with the NMR target being reached by poorer families 38 years later than the wealthiest families. Improved understanding of the factors that influence the NMR across the different wealth quintiles is essential to prioritize the interventions needed to improve neonatal survival and achieve the ENAP target by 2030.

These findings are consistent with several studies conducted in South Asia and Sub-Saharan Africa. A further analysis of Ghana’s Demographic and Health Surveys 2002 and 2008 showed, infants of grand multiparous mothers were more likely to die during the neonatal period, whereas adequate utilization of antenatal, delivery and postnatal health services (AOR 0.25, 95% CI 0.13, 0.46) reduced the likelihood of neonatal mortality. Families with high socioeconomic deprivation was associated with increased neonatal mortality (AOR 3.38, 95% CI 1.42, 8.04). (Kayode et al., 2014). A further analysis of Afghanistan’s demographic and health survey 2015 showed smaller than average size (AOR 1.8, 95% CI 1.2–2.6) and larger size babies (AOR 2.9, 95% CI 2.2–3.8) had higher risk for neonatal mortality than the average size baby. The study also showed women giving birth at or before 18 years of age ((AOR 1.8; 95% CI 1.1–3.2) and ≥ 35 years (AOR 1.7; 95% CI 1.3–2.3) had higher risk for neonatal mortality than 19–34 year women. Women with interpregnancy interval of less than 2 years (AOR 2.6, 95% CI 1.3–4.9) had risk for neonatal mortality than those who gave birth in more than 2-year interval (Kibria et al. 2015). A further analysis of 2013 Nigeria DHS showed a difference in neonatal mortality between the urban and rural population. The risk factors for neonatal mortality in urban population was lack of electricity (AOR 1.56; 95% CI 1.09–2.22), small birth size (AOR 3.05, 95% CI 2.05–4.54), and male gender (AOR 1.67, 95% CI 1.22–2.23) (Adewuyi and Zhao 2017). A further analysis of the 2012–2013 Pakistan DHS showed that employed mothers had higher odds of neonatal mortality (OR 1.47) while women living in consanguineous marriages had higher odds of infant mortality (OR 1.45) and under-five mortality (OR 1.38). Children in regions with disproportionately poor, rural with low levels of education, were at highest risk of dying (Helova et al. 2017).

Our study has several strengths. First, Nepal DHS has large sample sizes and higher response rates (almost 97%). Second, results from population-based study can be used for informed decision making for neonatal health programming. Third, the similar questionnaires were used to collect information in all four surveys which increases coherence in data analysis. Limitation to this study include a nationally representative cross-sectional survey to provide point estimates of neonatal mortality. A preferable source of data would have been vital registration statistics; but Nepal lacks a robust vital registration system. Vital registration provides more accurate estimates of neonatal mortality as well as risks and causes of neonatal mortality. The other major limitation was that NDHS data are from interviews with mothers about behaviours, which could be inaccurate due to recall and reporting bias.

The administration and funding of healthcare and development in general is being decentralized under Nepal’s new federal system of government as per the 2016 Constitution (Government of Nepal 2015). Ensuring multi-sectoral engagement for newborn health and survival at federal, provincial and local government levels is fundamental for implementing Nepal’s Every Newborn Action Plan. The engagement of the education, healthcare, water and sanitation sectors and of local communities and leaders for the development of their areas will go a long way to overcoming social determinant to neonatal health and survival. Ensuring accountability for maternal and neonatal care by strengthening maternal health services and referral care for newborn health through a quality improvement mechanism should sustain the improvements in health service delivery. Multi-sectoral action for reducing the inequity gap in neonatal survival will provide evidence on how financing maternal and neonatal health can be sustained through decentralized planning and budgeting.

The study showed many factors to be associated with neonatal mortality in Nepal. The continuation of the current trends in the neonatal mortality rate in different socioeconomic groups would widen the inequity gap by 2030. The reduction of neonatal mortality proportionately across the different socio-economic groups requires a multi-sectoral approach to address the causal factors.

## Electronic supplementary material

Below is the link to the electronic supplementary material.

Supplementary material 1 (DOCX 20 kb)

## Abbreviation

ENAP: Every Newborn Action Plan
NMR: Neonatal mortality rate
AOR: Adjusted odds ratio
MDG: Millennium development goals
SDG: Sustainable development goals
IMNCI: Integrated management of neonatal and childhood illness
NDHS: Nepal demographic and health survey
