# Supplementary material for: Trends for Neonatal Deaths in Nepal (2001–2016) to Project Progress Towards the SDG Target in 2030, and Risk Factor Analyses to Focus Action
Source: Matern Child Health J. 2019 Nov 26;24(Suppl 1):5–14. doi: 10.1007/s10995-019-02826-0 (PMC7048722; doi:10.1007/s10995-019-02826-0)
Supplement: Supplementary file 1 — Supplementary material 1 (DOCX 20 kb) [file 10995_2019_2826_MOESM1_ESM.docx]

Web-appendix

Table S-1 Sampling frame, domain used, sample size and response rate for 2001 to 2016 NDHSs

|  | 2001 NDHS | 2006 NDHS | 2011 NDHS | 2016 NDHS |
| --- | --- | --- | --- | --- |
| Sampling frame | 1991 census | 2001 census | 2001 census | 2011 census |
| Sample Domain | 13 | 13 | 13 | 14 |
| Sample size | 8,700 households  8,400 women | 9,036 households 8,600 women | 11,085 households  13,485 women | 11,473 households  13,089 women |
| Response rate | 98% women  96% men | 98% women  96% men | 98% women  95% men | 99% women  96% men |

Table S-2. The trend in neonatal mortality rate by socio-demographic characteristics (per 1000 live birth, 2001–2016 NDHSs)

|  | 2001 | 2006 | 2011 | 2016 |
| --- | --- | --- | --- | --- |
| Neonatal mortality rate | 39 | 33 | 33 | 21 |
| **Wealth quintile** |  |  |  |  |
| Poorest | 49 | 43 | 37 | 36 |
| Second poorest | 56 | 38 | 40 | 33 |
| Middle | 49 | 47 | 39 | 26 |
| Second richest | 47 | 31 | 37 | 20 |
| Richest | 32 | 26 | 19 | 12 |
| **Residence** |  |  |  |  |
| Urban | 37 | 25 | 25 | 21 |
| Rural | 49 | 40 | 36 | 33 |
| **Mother’s level of education** |  |  |  |  |
| No education | 50 | 42 | 39 | 33 |
| Primary education or more | 25 | 21 | 26 | 16 |
| **Maternal age in completed years** |  |  |  |  |
| <20 | 71 | 55 | 51 | 39 |
| 20-29 | 40 | 32 | 32 | 21 |
| 30-39 | 43 | 36 | 27 | 31 |
| 40-49 | 80 | 38 | 29 | 51 |
| **Interval since previous birth** |  |  |  |  |
| >48 months | 80 | 40 | 26 | 22 |
| 36-48 months | 63 | 31 | 19 | 20 |
| 24-36 months | 57 | 31 | 21 | 14 |
| <24 months | 46 | 22 | 22 | 12 |
| **Birth order of infant** |  |  |  |  |
| 1 | 58 | 47 | 45 | 30 |
| 2 | 44 | 29 | 30 | 19 |
| 3 | 39 | 39 | 31 | 29 |
| 4 | 63 | 52 | 41 | 99 |
| **Sex of infant** |  |  |  |  |
| Male | 52 | 39 | 37 | 33 |
| Female | 43 | 37 | 33 | 20 |
| **Size of infant at birth** |  |  |  |  |
| Average or larger | 32 | 27 | 29 | 17 |
| Small or very small | 58 | 56 | 51 | 35 |

Table S-3. Projection of neonatal mortality rate per 1000 live birth (2016-2067)

| **Year** | **Lowest** | **Second** | **Middle** | **Fourth** | **Highest** | **Average** |
| --- | --- | --- | --- | --- | --- | --- |
| Yr 2016 | 36 | 33 | 26 | 20 | 12 | 21 |
| Yr 2017 | 35 | 32 | 25 | 19 | 11 | 20 |
| Yr 2018 | 35 | 32 | 24 | 18 | 10 | 19 |
| Yr 2019 | 34 | 31 | 23 | 17 | 10 | 18 |
| Yr 2020 | 33 | 30 | 22 | 16 | 9 | 18 |
| Yr 2021 | 32 | 30 | 21 | 15 | 8 | 17 |
| Yr 2022 | 32 | 29 | 20 | 14 | 8 | 16 |
| Yr 2023 | 31 | 28 | 19 | 13 | 7 | 16 |
| Yr 2024 | 30 | 28 | 18 | 12 | 7 | 15 |
| Yr 2025 | 30 | 27 | 17 | 12 | 6 | 14 |
| Yr 2026 | 29 | 27 | 17 | 11 | 6 | 14 |
| Yr 2027 | 29 | 26 | 16 | 10 | 6 | 13 |
| Yr 2028 | 28 | 26 | 15 | 10 | 5 | 13 |
| Yr2029 | 27 | 25 | 15 | 9 | 5 | 12 |
| **Yr 2030** | **27** | **25** | **14** | **9** | **5** | **11** |
| Yr 2031 | 26 | 24 | 13 | 8 | 5 | 11 |
| Yr 2032 | 26 | 24 | 13 | 8 | 5 | 11 |
| Yr 2033 | 25 | 23 | 12 | 7 | 5 | 10 |
| Yr 2034 | 25 | 23 | 12 | 7 | 5 | 10 |
| Yr 2035 | 24 | 22 | 11 | 6 | 5 | 9 |
| Yr 2036 | 24 | 22 | 11 | 6 | 5 | 9 |
| Yr 2037 | 23 | 21 | 10 | 6 | 5 | 9 |
| Yr 2038 | 23 | 21 | 10 | 5 | 5 | 8 |
| Yr 2039 | 22 | 20 | 9 | 5 | 5 | 8 |
| Yr 2040 | 22 | 20 | 9 | 5 | 5 | 7 |
| Yr 2041 | 21 | 20 | 9 | 5 | 5 | 7 |
| Yr 2042 | 21 | 19 | 8 | 5 | 5 | 7 |
| Yr 2043 | 20 | 19 | 8 | 5 | 5 | 7 |
| Yr 2044 | 20 | 18 | 8 | 5 | 5 | 6 |
| Yr 2045 | 20 | 18 | 7 | 5 | 5 | 6 |
| Yr 2046 | 19 | 18 | 7 | 5 | 5 | 6 |
| Yr 2047 | 19 | 17 | 7 | 5 | 5 | 6 |
| Yr 2048 | 18 | 17 | 6 | 5 | 5 | 5 |
| Yr 2049 | 18 | 17 | 6 | 5 | 5 | 5 |
| Yr 2050 | 18 | 16 | 6 | 5 | 5 | 5 |
| Yr 2051 | 17 | 16 | 6 | 5 | 5 | 5 |
| Yr 2052 | 17 | 16 | 5 | 5 | 5 | 5 |
| Yr 2053 | 17 | 15 | 5 | 5 | 5 | 5 |
| Yr 2054 | 16 | 15 | 5 | 5 | 5 | 5 |
| Yr 2055 | 16 | 15 | 5 | 5 | 5 | 5 |
| Yr 2056 | 16 | 14 | 5 | 5 | 5 | 5 |
| Yr 2057 | 15 | 14 | 5 | 5 | 5 | 5 |
| Yr 2058 | 15 | 14 | 5 | 5 | 5 | 5 |
| Yr 2059 | 15 | 13 | 5 | 5 | 5 | 5 |
| Yr 2060 | 14 | 13 | 5 | 5 | 5 | 5 |
| Yr 2061 | 14 | 13 | 5 | 5 | 5 | 5 |
| Yr 2062 | 14 | 13 | 5 | 5 | 5 | 5 |
| Yr 2063 | 13 | 12 | 5 | 5 | 5 | 5 |
| Yr 2064 | 13 | 12 | 5 | 5 | 5 | 5 |
| Yr 2065 | 13 | 12 | 5 | 5 | 5 | 5 |
| Yr 2066 | 13 | 12 | 5 | 5 | 5 | 5 |
| Yr 2067 | 12 | 11 | 5 | 5 | 5 | 5 |
